# Global trends and hotspots of randomized controlled trials in chronic pain therapy (1989–2024): A 35-year bibliometric analysis

**DOI:** 10.1097/MD.0000000000043936

**Published:** 2025-09-26

**Authors:** Meng Yang, Liuxue Lu, Yingge Tong, Qingye Liang, Qiuhuan Huang, Yuhai Yang, Hanxiang Li

**Affiliations:** a Affiliated Hospital of Youjiang Medical University for Nationalities, Key Laboratory of Molecular Pathology for Hepatobiliary Diseases of Guangxi, Baise, China; b Nursing College, Hangzhou Normal University, Hangzhou, China; c Nursing College, Youjiang Medical University for Nationalities, Baise, China.

**Keywords:** bibliometric analysis, chronic pain, CiteSpace, RCTs, therapy, VOSviewer

## Abstract

**Background:**

Chronic pain persists as a significant global health challenge, underscoring the necessity for effective, evidence-based treatment strategies. This study aims to assess publication trends and identify research hotspots within randomized controlled trials (RCTs) pertaining to chronic pain therapy.

**Methods:**

We conducted a bibliometric analysis utilizing data from Web of Science Core Collection, covering the period from January 1989 to September 2024. Eligible studies were extracted from, and analysis was performed by VOSviewer, CiteSpace, and R 4.3.3.

**Results:**

Our analysis encompassed a total of 4206 publications sourced from 939 journals, authored by 20,068 individuals across 86 countries. The most cited document was “*Clinical importance of changes in chronic pain intensity measured on an 11-point numerical pain rating scale*.” The USA led in both publication volume and citation frequency, followed by the UK and Canada. Harvard University emerged as the most prolific institution, with significant contributions from journals such as *Pain* and *Pain Medicine*. Notable authors included Moore R. Andrew and Manchikanti Laxmaiah. Keyword analysis revealed research hotspots in “low back pain,” “management,” and “double blind,” while emerging research frontiers included “guidelines” and “hip,” indicating areas for future inquiry.

**Conclusion:**

This bibliometric analysis of RCTs pertaining to chronic pain therapy elucidated trends and emergent themes within the field. The findings yield significant insights for clinical applications and serve as a reference for prospective research directions in chronic pain management. This analysis reveals a paradigm shift from pharmacological trials to multimodal interventions incorporating psychological outcomes.

## 1. Introduction

Chronic pain constitutes a significant global health challenge, affecting 30% to 50% of adults in specific regions, particularly within aging populations and in low- and middle-income countries (LMICs).^[1]^ The ramifications of chronic pain extend beyond physical discomfort, resulting in functional disabilities, a reduced quality of life, and substantial economic losses attributed to decreased productivity and increased healthcare utilization.^[2]^ Moreover, chronic pain is frequently undertreated and often coexists with psychiatric conditions such as depression and anxiety, fostering a bidirectional relationship that exacerbates individual suffering and imposes additional burdens on healthcare systems.^[3]^

Randomized controlled trials (RCTs) are critical for evaluating interventions for chronic pain, which encompass pharmacological treatments, psychological therapies, and multimodal approaches.^[4,5]^ Meta-analytical evidence substantiates the effectiveness of interventions such as cognitive behavioral therapy (CBT) and mindfulness-based programs for managing conditions like chronic low back pain.^[6]^ However, a notable paradox arises: despite the increasing prevalence of chronic pain in LMICs, the volume of RCT outputs from these regions remains disproportionately low, impeded by systemic barriers including inadequate funding and limited research infrastructure.^[7]^ This geographic disparity raises concerns regarding the applicability of existing evidence to populations experiencing a high burden of chronic pain.

Bibliometric analysis, a quantitative method for mapping scholarly output, offers valuable insights into research trends by examining publication dynamics, collaboration networks, and knowledge gaps.^[8–10]^ While systematic reviews have synthesized the efficacy of various interventions,^[6]^ few studies have employed bibliometric methods to investigate RCT trends in chronic pain research. Recent finding reveal that nearly 30% of registered chronic pain RCTs remain unpublished or are discontinued,^[11]^ highlighting inefficiencies in knowledge translation. Such publication bias and geographic imbalances may perpetuate redundant studies while neglecting underserved populations. To address these gaps, this study conducts a 35-year bibliometric analysis (1989–2024) of RCTs targeting chronic pain therapies. We aim to: Quantify global publication trends and identify geographic/institutional contributors; map thematic shifts and emerging intervention modalities; evaluate disparities between research focus areas and disease burden patterns. By linking bibliometric patterns to clinical epidemiology, this work seeks to inform strategic resource allocation and prioritize RCTs addressing populations with the greatest unmet needs, particularly in LMICs.

## 2. Materials and methods

### 2.1. Search strategies and data collection

A comprehensive literature search was conducted utilizing the Web of Science Core Collection (WoSCC) to identify studies published between 1989 and 2024. The WoSCC includes over 12,000 scholarly journals, rendering it a more reliable source for bibliometric analysis compared to other databases such as Scopus, Medline, and PubMed. The search strategy employed the following formula: TS = (“chronic pain”) AND (((((TS = (treatment)) OR TS = (treated)) OR TS = (therapy)) OR TS = (treat)) OR TS = (therapeutic)) OR TS = (cure)) AND ((((((((TS = (“randomized controlled trial”)) OR TS = (“randomized controlled trials”)) OR TS = (“randomized control trial”)) OR TS = (“randomized controlled trial”)) OR TS = (“randomized clinical trial”)) OR TS = (“randomized controlled clinical trial”)) OR TS = (“randomized controlled study”)) OR TS = (randomized clinical trials)) OR TS = (RCT*). The analysis was restricted to articles published in English. To ensure consistency in light of potential database updates, literature retrieval was performed on a single day, September 20, 2024. Data collected in text format included the number of publications and citations, titles, author details, institutions, countries/regions, keywords, and journals, for subsequent bibliometric analysis.

### 2.2. Statistical analysis and visualization

The bibliometric analysis employed a range of tools, including Microsoft Excel, VOSviewer 1.6.20, CiteSpace 6.3.R1, and R 4.3.3. VOSviewer is a versatile software application that facilitates the mapping of institutional collaborations, author collaborations, co-authorships, citations, and co-citations. Through the application of VOSviewer, co-occurrence analysis was utilized to elucidate the relationships among various keywords.

To identify emerging trends and research hotspots, CiteSpace was employed to analyze burst keywords, with the parameters configured for time slicing from January 1994 to September 2024, utilizing a 1-year interval and keyword node types. The threshold for keyword inclusion was established at the top 5 per segment, and the pruning method applied was a combination of pathfinder and pruning merge networks. This process culminated in the creation of a visual analysis, resulting in a keyword timeline graph pertinent to RCTs focused on chronic pain therapy. Moreover, R 4.3.3 offers robust bibliometric analysis capabilities, including the construction of bibliographic coupling, co-citation, and co-authorship networks. This tool supports the importation, transformation, analysis, and visualization of data. Microsoft Excel was employed to compute bibliometric metrics such as annual publication counts, citation frequencies, journal impact factors, and author statistics. Journals were categorized into quartiles based on their impact factors, as reported in the 2023 Journal Citation Reports. Utilizing this data, the H-index, M-index, and G-index were calculated to quantify the academic impact of individuals and journals. The H-index, the most widely utilized bibliometric metric, is defined as the number of papers (H) that have received a minimum of H citations. The G-index enhances the H-index by evaluating the global citation performance of a set of articles, while the M-index denotes the average number of citations received by the papers contributing to the H-index. In this study, the H-index for each author was derived from the WoSCC.

## 3. Results

### 3.1. An overview of publications

**Figure 1.**
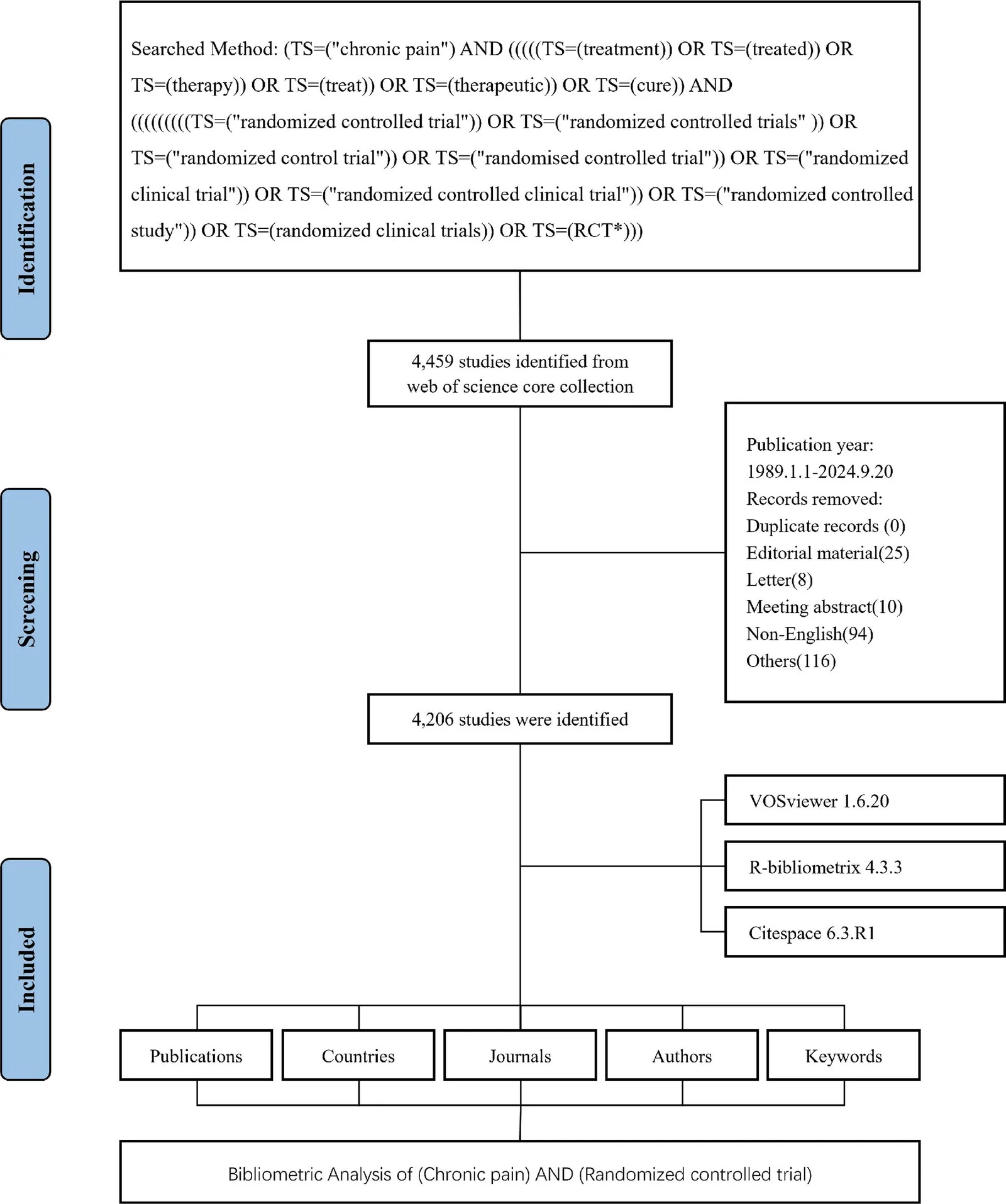
Flowchart of the literature screening process.

**Figure 2.**
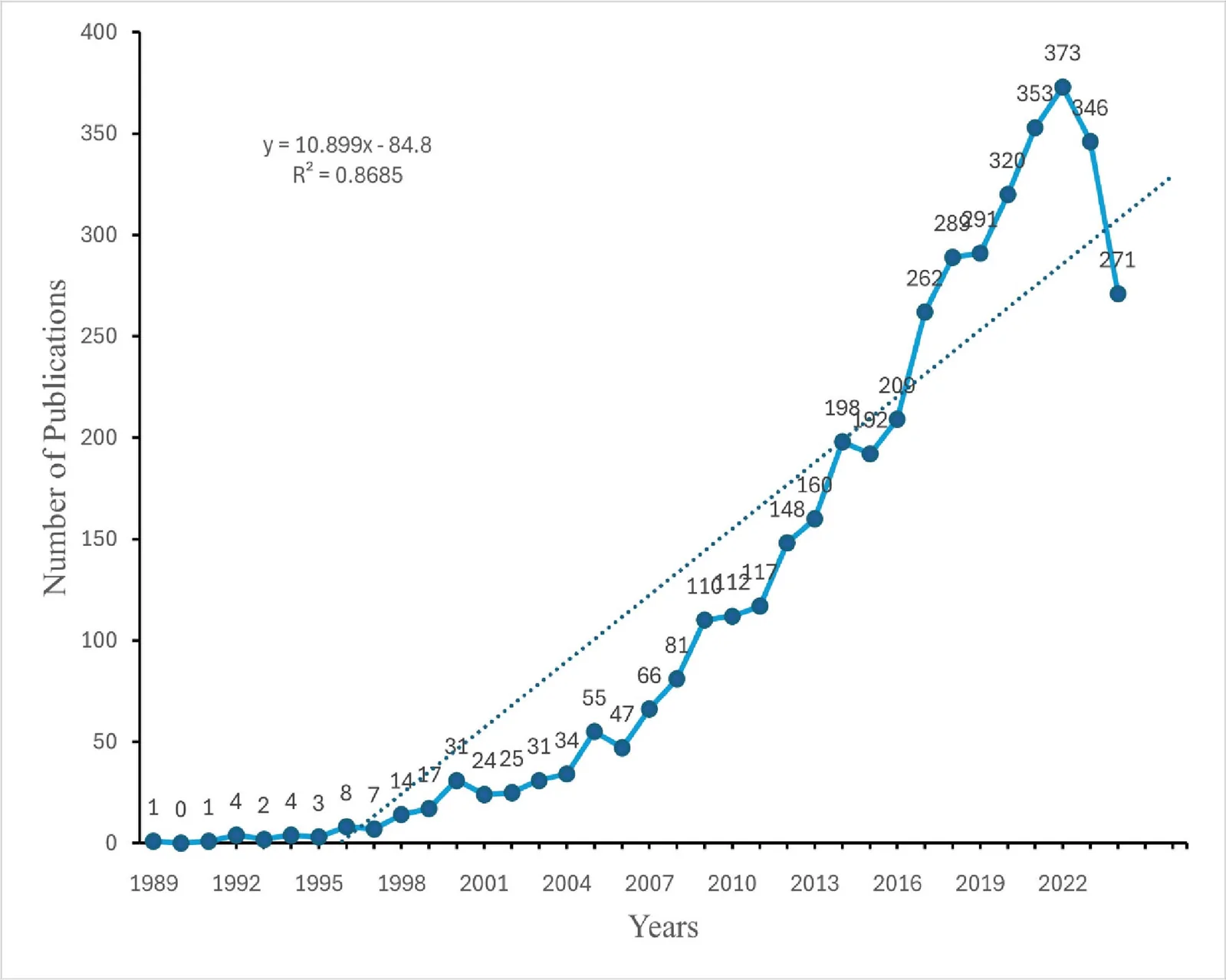
Growth trend of the publications worldwide.

The bibliometric analysis of research on randomized controlled trials (RCTs) related to chronic pain therapy from 1989 to 2024, as illustrated in the literature screening flow chart (Fig. 1), initially identified 4459 records. After excluding 253 non-article types, we retained 4206 eligible publications from 939 sources. This analysis included contributions from 20,068 authors affiliated with 14,553 institutions across 86 countries, with studies published in 939 journals and collectively citing a total of 130,129 works. The publication trend remained relatively flat with minimal outputs until 1998, when a steady increase began in the late 1990s. A notable surge occurred starting in 1999, which saw an additional 14 articles published that year. The year 2017 marked significant growth, with 53 articles published. The total number of publications peaked at 373 in 2022, indicating a robust and growing interest in this research area. To further assess trends in RCTs of chronic pain therapy, we developed a predictive equation for cumulative publication count (*Y*) based on publication year (*x*): *Y* = 10.899*x* − 84.8. The *R*-squared value for predicting the number of annual publications was 86.85% (*R*^2^ = 0.8685) (Fig. 2).

### 3.2. Country/region analysis

**Figure 3.**
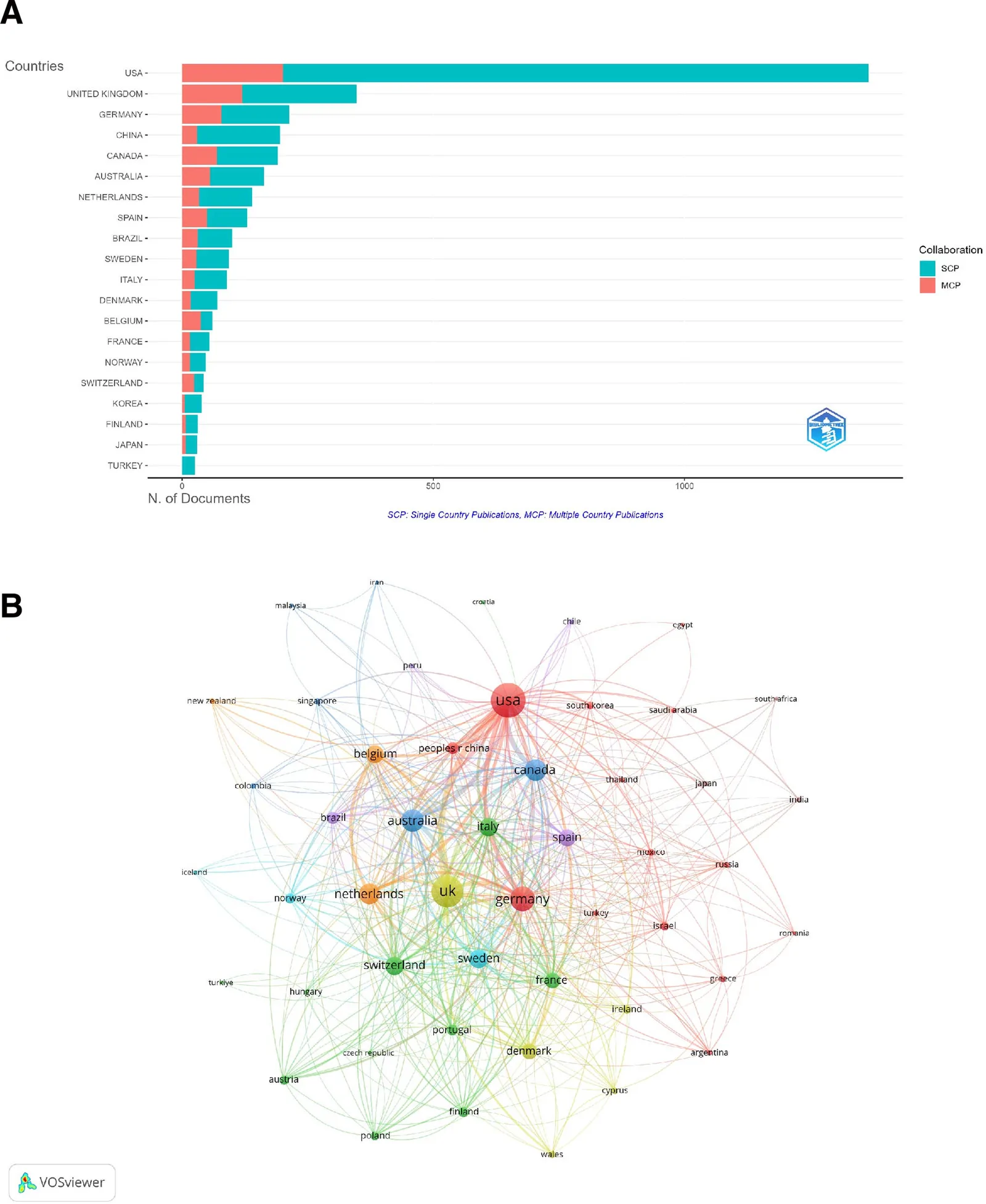
Analysis of countries. (A) Distribution of corresponding author’s publications by country. (B) Visualization map depicting the collaboration among different countries.

A total of 86 countries have contributed to the literature in this field. The top 20 producing countries accounted for 3425 articles, representing 81.4% of the global output, with contributions from Asia, North America, Europe, and Oceania. The USA led in productivity, publishing the highest number of articles at 7049, followed by the UK with 1506 and Canada with 1076. In terms of citation impact, the USA also ranked first, with a total of 69,908 citations, followed by the UK (22,603), Germany (10,971), and Australia (7453) (Table 1). Belgium had the highest ratio of multiple country publications at 61.7%, followed by Switzerland at 55.8% and Spain at 38.5% (Fig. 3A). An analysis using VOSviewer was conducted on 49 countries that produced at least 5 coauthored documents (Fig. 3B). China demonstrated the highest level of international collaboration, engaging in 782 partnerships with other countries, followed by the UK with 654 collaborations and Germany with 412.

**Table 1:** Publication and citation profiles of leading countries.

Country	Articles	Freq	MCP ratio	TP	TP rank	TC	TC rank	Average citations
USA	1366	0.325	0.147	7049	1	69,908	1	51.2
UK	347	0.083	0.343	1506	2	22,603	2	65.1
Germany	213	0.051	0.366	837	4	10,971	3	51.5
China	195	0.046	0.154	737	5	2563	9	13.1
Canada	190	0.045	0.363	1074	3	7114	6	37.4
Australia	163	0.039	0.344	711	6	7453	4	45.7
Netherlands	139	0.033	0.245	654	8	7209	5	51.9
Spain	130	0.031	0.385	672	7	2408	10	18.5
Brazil	100	0.024	0.310	397	10	1669	14	16.7
Sweden	93	0.022	0.301	430	9	3328	8	35.8

Articles = publications of corresponding authors only, Average citations = the average number of citations per publication, Freq = frequence of total publications, MCP ratio = proportion of multiple country publications, TC = total citations, TC rank = rank of total citations, TP = total publications, TP rank = rank of total Publications.

### 3.3. Institutions analysis

**Figure 4.**
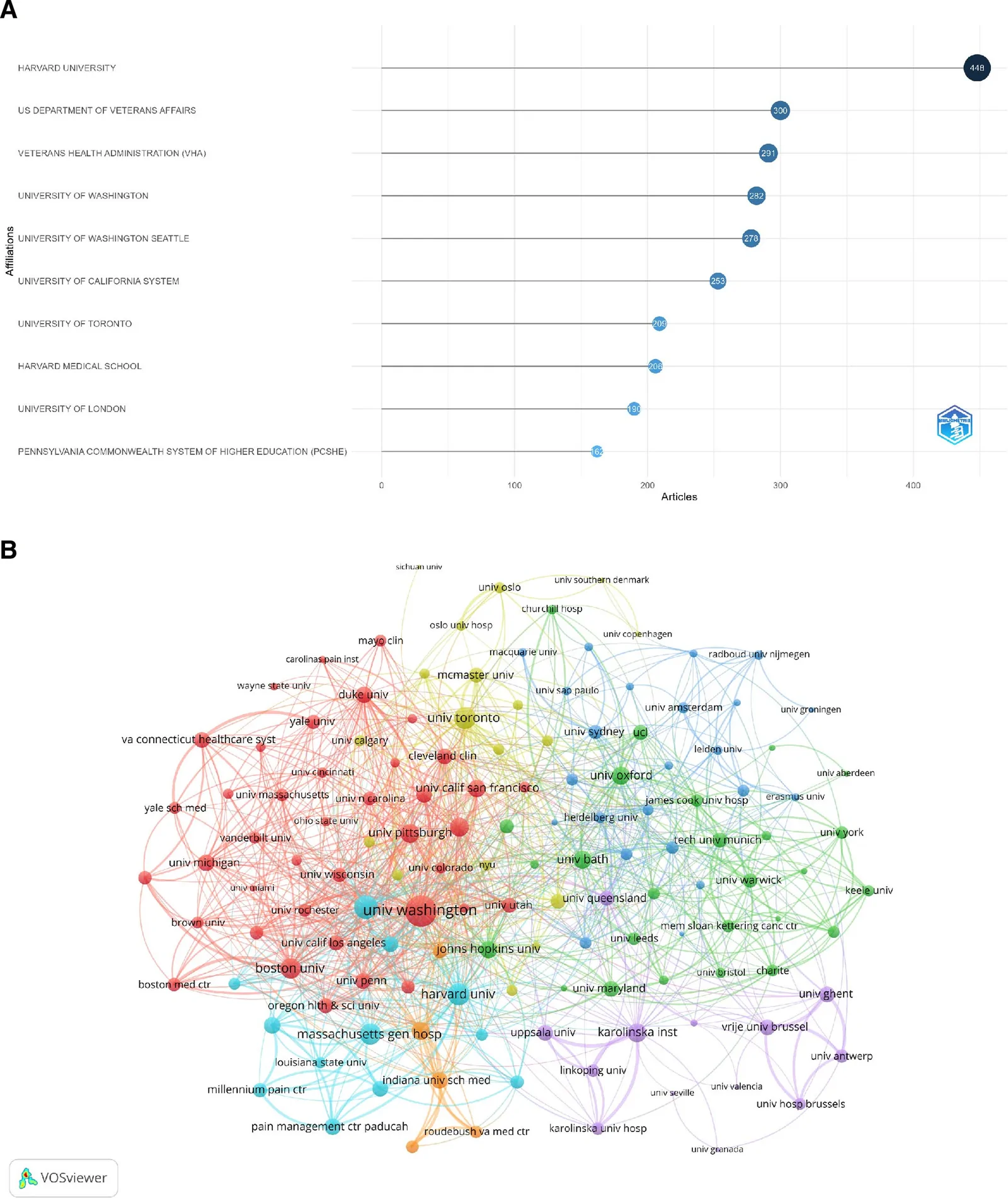
Analysis of institutions. (A) Top 10 institutions by article count and rank. (B) Visualization networks of institution collaborations.

Figure 4A shows the 10 institutions that have made the most substantial contributions to the academic literature. Harvard University ranks first with 448 published articles, followed by the U.S. Department of Veterans Affairs with 300 articles and the Veterans Health Administration with 291 articles. Other significant contributors include the University of Washington (282 articles), University of Washington Seattle (278 articles), and the University of California System (253 articles). Additionally, Figure 4B illustrates the collaborative relationships among 137 institutions, each exhibiting a total link strength of at least 15. Three key cooperative groups stand out, showcasing the strongest partnerships. The University of Washington excels in international collaborations, totaling 283, while Harvard Medical School and Harvard University contribute 159 and 144 collaborations, respectively.

### 3.4. Journals analysis

Table 2 presents the characteristics of the 20 most productive journals that have published literature on RCTs related to chronic pain therapy in recent years. These journals collectively contributed 1564 studies, representing 37.2% of all retrieved publications in this field. High-impact journals, such as *PAIN* and *Pain Medicine*, made the most significant contributions. *PAIN* (IF = 5.9; H-index = 71; total publications = 206) recorded the highest number of publications and ranked first in total citations, with 14,746, followed by *Pain Medicine* (IF = 2.9; H-index = 33; total publications = 143), which ranked second in publications and fifth in citations, with 3170. *The Cochrane Database of Systematic Reviews* (IF = 8.8; H-index = 60; total publications = 139) ranked third in publications. Other notable contributors include the *Journal of Pain* (IF = 4.0; H-index = 31; total publications = 96), which ranked second in citations at 4125, and the *Clinical Journal of Pain* (IF = 2.6; H-index = 41; total publications = 107), which ranked 4th in citations with 3643. Additionally, *SPINE* (IF = 2.6; H-index = 17) had a notable impact with 3863 citations. Most top-ranking journals are classified in the top 2 quartiles of the journal citation reports, with *PAIN* and *The Cochrane Database of Systematic Reviews* in Q1, indicating their high influence. Conversely, journals like *Pain Physician* (Q2) and *BMC Musculoskeletal Disorders* (Q3) maintain significant contributions within their specialized areas.

**Table 2:** Bibliometric indicators of high-impact journals.

Journal	H-index	IF	JCR Quartile	PY start	TP	TP rank	TC	TC rank
*PAIN*	71	5.9	Q1	1991	206	1	14,746	1
*Cochrane Database of Systematic Reviews*	60	8.8	Q1	2005	139	3	3112	6
*Pain Physician*	43	2.6	Q2	2008	110	5	3088	7
*Clinical Journal of Pain*	41	2.6	Q2	1994	107	6	3643	4
*Pain Medicine*	33	2.9	Q1	2000	143	2	3170	5
*Journal of Pain*	31	4	Q2	2000	96	8	4125	2
*European Journal of Pain*	30	3.5	Q2	2001	78	10	2635	8
*Neuromodulation*	23	3.2	Q2	2000	60	11	1819	12
*Journal of Pain Research*	21	2.5	Q2	2013	81	9	799	34
*BMC Musculoskeletal Disorders*	18	2.2	Q3	2003	58	12	876	29

Average citations = the average number of citations per publication, H-index = The h-index of the journal, which measures both the productivity and citation impact of the publications, IF = impact factor, indicating the average number of citations to recent articles published in the journal, JCR quartile = the quartile ranking of the journal in the Journal Citation Reports, indicating the journal’s ranking relative to others in the same field (Q1: top 25%, Q2: 25%–50%, Q3: 50%–75%, Q4: bottom 25%). PY start = publication year start, indicating the year the journal started publication, TC = total citations, TC rank = rank of total citations, TP = total publications, TP rank = rank of total publications.

**Figure 5.**
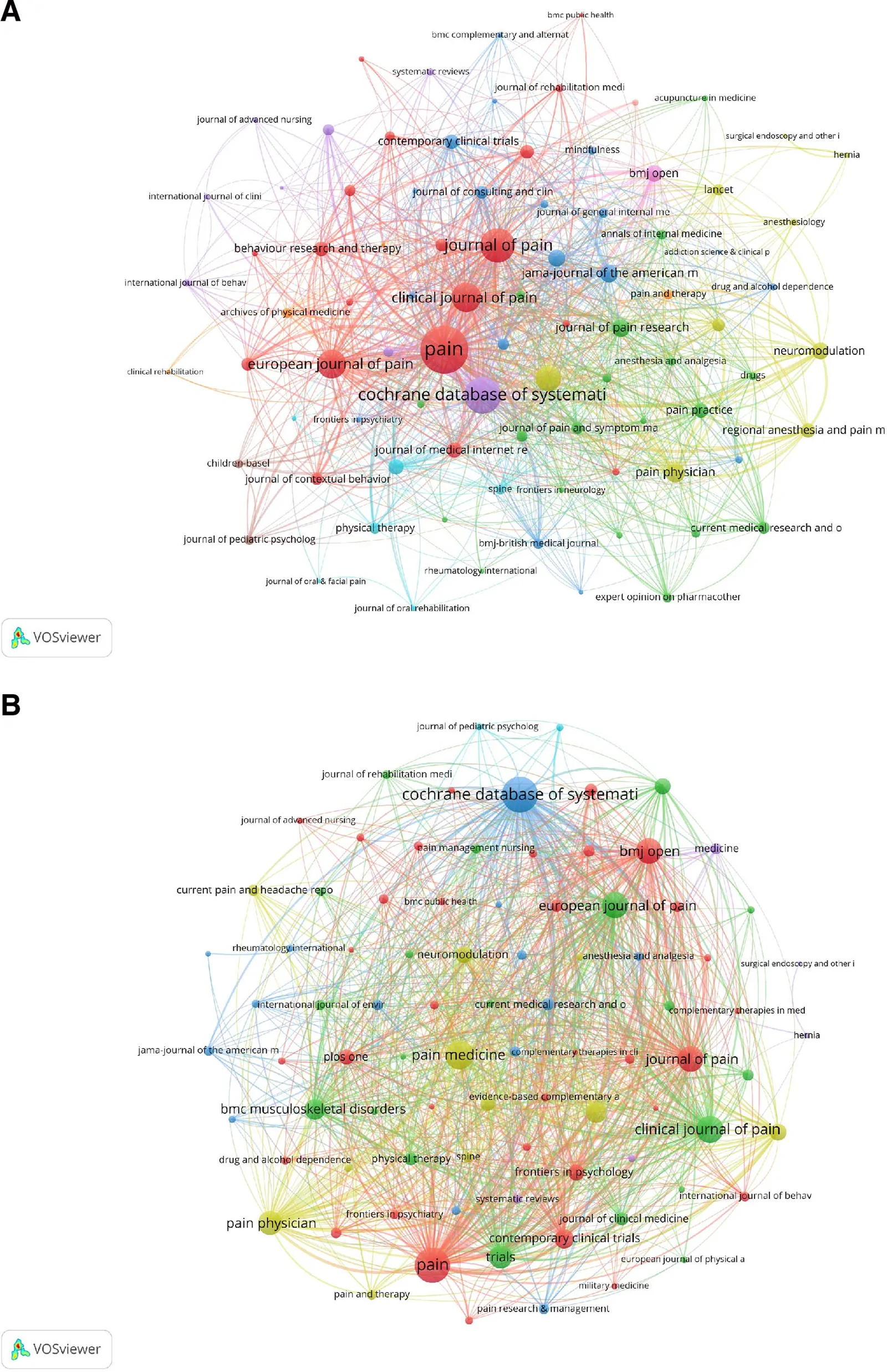
Analysis of journals. (A) Visualization map depicting the co-occurrence network. (B) Visualization map depicting the coupling network.

The network visualization map from the journal co-citation analysis revealed co-occurrence networks comprising 87 journals, each with at least 8 occurrences (Fig. 5A). The 3 key journals with the highest total link strength in the co-occurrence networks were *PAIN* (1565), *The Cochrane Database of Systematic Reviews* (910), and the *Journal of Pain* (817). Similarly, the coupling networks included 87 journals with at least 8 connections (Fig. 5B). The leading journals with the highest total link strength in the coupling networks were *The Cochrane Database of Systematic Reviews* (64,220), *PAIN* (62,569), and *Pain Medicine* (41,402).

### 3.5. Authors analysis

**Figure 6.**
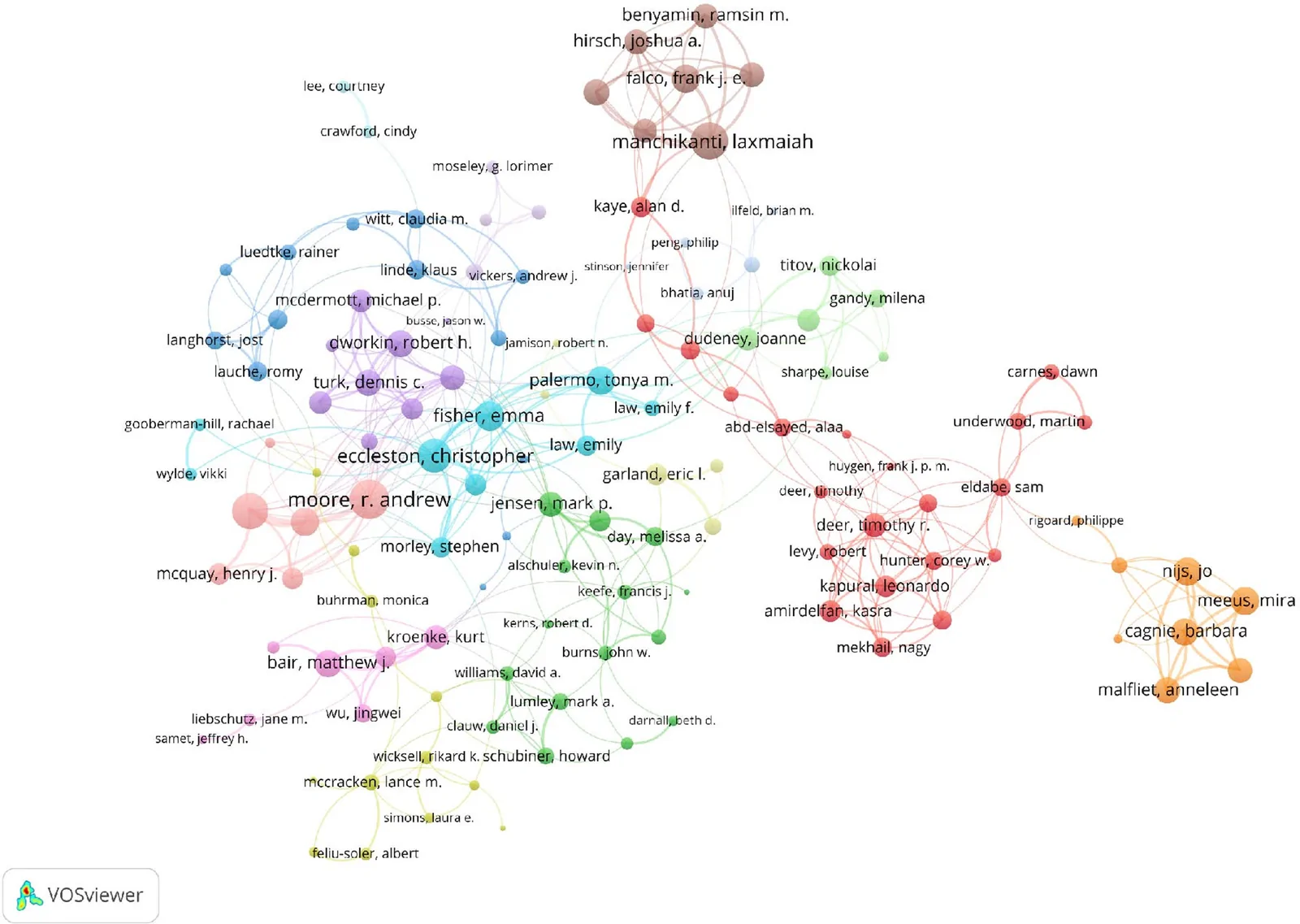
Visualization map depicting the collaboration among different authors.

A total of 20,068 authors contributed to publications within this field. The top 20 most influential authors are listed in Table 3. Moore R. Andrew emerges as the most influential author, holding the highest H-index of 29 and a cumulative total of 3744 citations, while also ranking first with 44 publications. He shares the top H-index position with Manchikanti Laxmaiah, who has an M-index of 1.71 and ranks third in total publications (37) with 2845 citations. Derry Sheena follows closely with an H-index of 25 and an M-index of 1.47, contributing 35 publications and ranking 4th in total citations (2406). Additionally, Eccleston Christopher is notable for amassing 3497 citations. The coauthor network engaged in RCTs of chronic pain therapy is divided into 11 groups (Fig. 6). Among the 129 authors involved in international collaborations with at least 8 articles, Moore R. Andrew leads with 94 collaborations, followed by Manchikanti Laxmaiah (81) and Derry Sheena (80).

**Table 3:** Publication and citation profiles of high-impact authors.

Authors	H-index	G-index	M-index	PY_start	TP	TP_frac	TP_rank	TC	TC rank
Manchikanti Laxmaiah	29	37	1.71	2008	37	5.97	3	2845	3
Moore R. Andrew	29	44	1.71	2008	44	9.63	1	3744	1
Derry Sheena	25	35	1.47	2008	35	8.35	5	2406	4
Eccleston Christopher	22	33	1.29	2008	33	5.55	7	3497	2
Wiffen Philip J.	22	23	1.38	2009	23	4.85	14	2260	7
Palermo Tonya M.	20	37	1.25	2009	37	7.09	4	1652	15
Deer Timothy R.	18	28	1.20	2010	28	2.93	10	1743	13
Nijs Jo	17	24	1.06	2009	24	3.65	13	1236	22
Singh Vijay	17	17	1.00	2008	17	2.42	29	1639	16
Fisher Emma	16	21	1.23	2012	21	3.50	17	1344	19

Average citations = the average number of citations per publication, G-index = the g-index of the journal, which gives more weight to highly-cited articles, H-index = The h-index of the journal, which measures both the productivity and citation impact of the publications, M-index = the m-index of the journal, which is the h-index divided by the number of years since the first published paper, PY start = publication year start, indicating the year the journal started publication, TC = total citations, TC rank = rank of total citations, TP = total publications, TP rank = rank of total publications.

### 3.6. Keywords analysis

**Figure 7.**
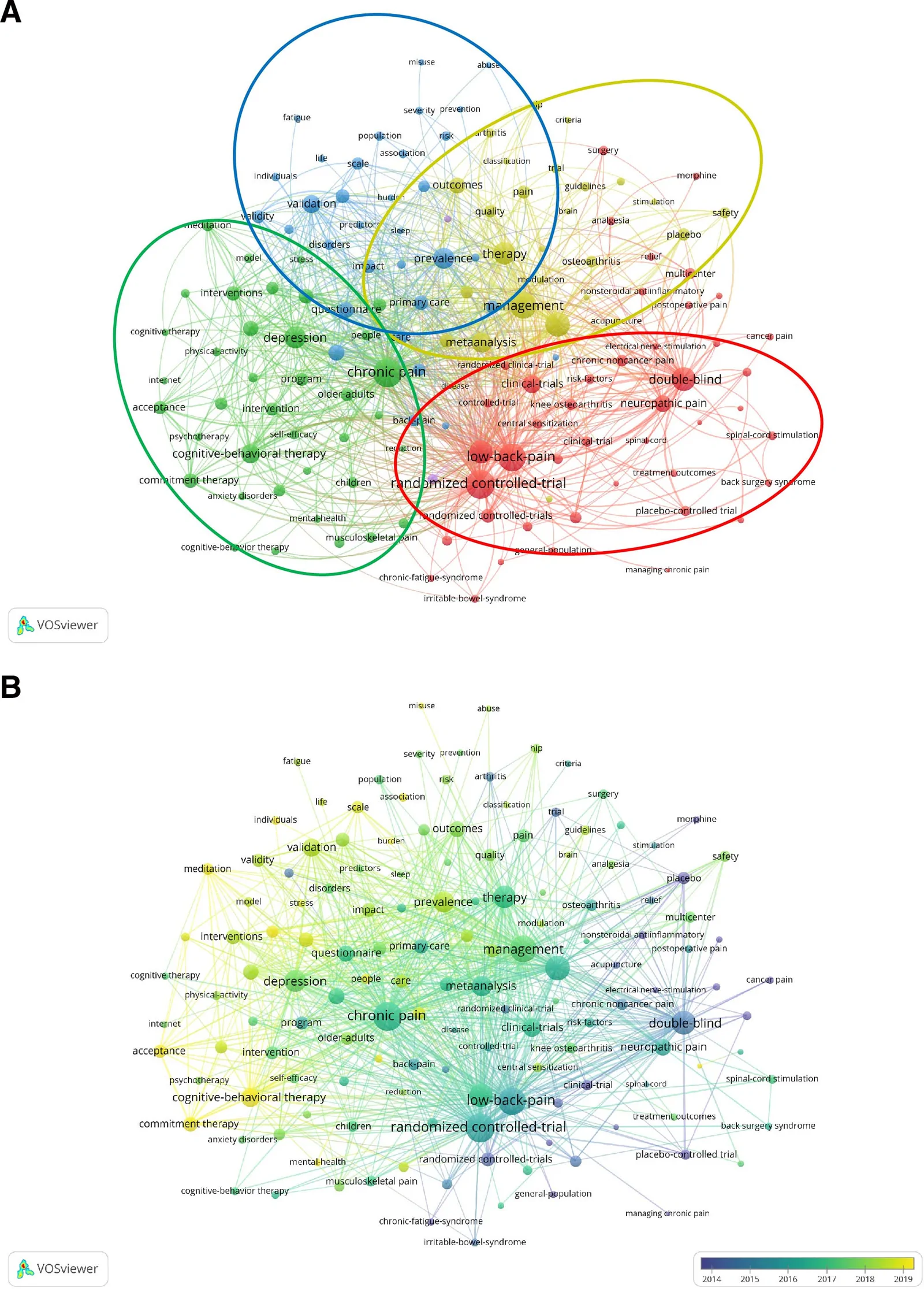
(A) Keyword cluster analysis. (B) Time trend analysis of the keyword co-occurrence network.

A comprehensive keyword analysis of the selected articles was conducted using the VOSviewer application. In total, 144 keywords were identified, each with at least 30 occurrences. The top 20 keywords based on frequency are presented in Table 4. Among these, the top 5 keywords unrelated to the direct theme were: “low back pain” (550), “management” (526), “double blind” (485), “efficacy” (431), and “quality of life” (389). In terms of total link strength, “low back pain” ranked highest (2152), followed closely by “management” (2135) and “efficacy” (1809). The keyword clustering analysis in RCTs focused on chronic pain therapy reveals 4 primary research themes (Fig. 7A). The red cluster addresses various types of pain and their specific treatment methods, including “low back pain,” “cancer pain,” and “postoperative pain,” with a strong emphasis on interventions like “double-blind trials” and “electrical nerve stimulation.” The green cluster focuses on psychological interventions and pain management strategies, incorporating keywords such as “acceptance,” “cognitive-behavioral therapy,” and “mindfulness.” The blue cluster pertains to pain assessment and individual factors, highlighting tools like “scales” and “risk” assessments to quantify the impact of chronic pain therapy on quality of life. The yellow cluster relates to alternative therapies and joint pain management, featuring keywords such as “acupuncture” and “osteoarthritis.” Lastly, the smaller purple cluster encompasses themes of “persistent pain” and “rehabilitation,” emphasizing the necessity for continuous care in chronic pain therapy. Additionally, the co-occurrence network reflecting keyword changes over time is presented in Figure 7B. From 2014 to 2016, keywords such as “double blind,” “clinical trial,” and “placebo” demonstrated significant influence, represented by large blue-green nodes. From 2016 to 2018, keywords like “therapy,” “depression,” “management,” and “meta-analysis” gained prominence, indicating a shift towards comprehensive chronic pain therapy and systematic analysis. During this period, “low back pain” also emerged as a large blue-green node, reflecting increased attention to specific types of pain. From 2018 onward, keywords such as “commitment therapy,” “cognitive-behavioral therapy,” and “acceptance” emerged as yellow nodes, signaling a growing interest in patient experiences and psychological interventions.

**Table 4:** The 20 most frequent keywords from 1995 to 2024.

Keyword	Occurrences	Total link strength
Chronic pain	661	2448
Randomized controlled-trial	654	2383
Low back pain	550	2152
Management	526	2135
Efficacy	431	1809
Double-blind	485	1670
Quality of life	389	1564
Therapy	346	1459
Depression	282	1378
Prevalence	286	1273
Meta-analysis	265	1162
Cognitive behavioral therapy	217	1036
Clinical trials	236	981
Validation	208	926
Neuropathic pain	240	842
Outcomes	181	770
Disability	157	737
Questionnaire	165	718
Anxiety	142	669
Health	147	642

**Figure 8.**
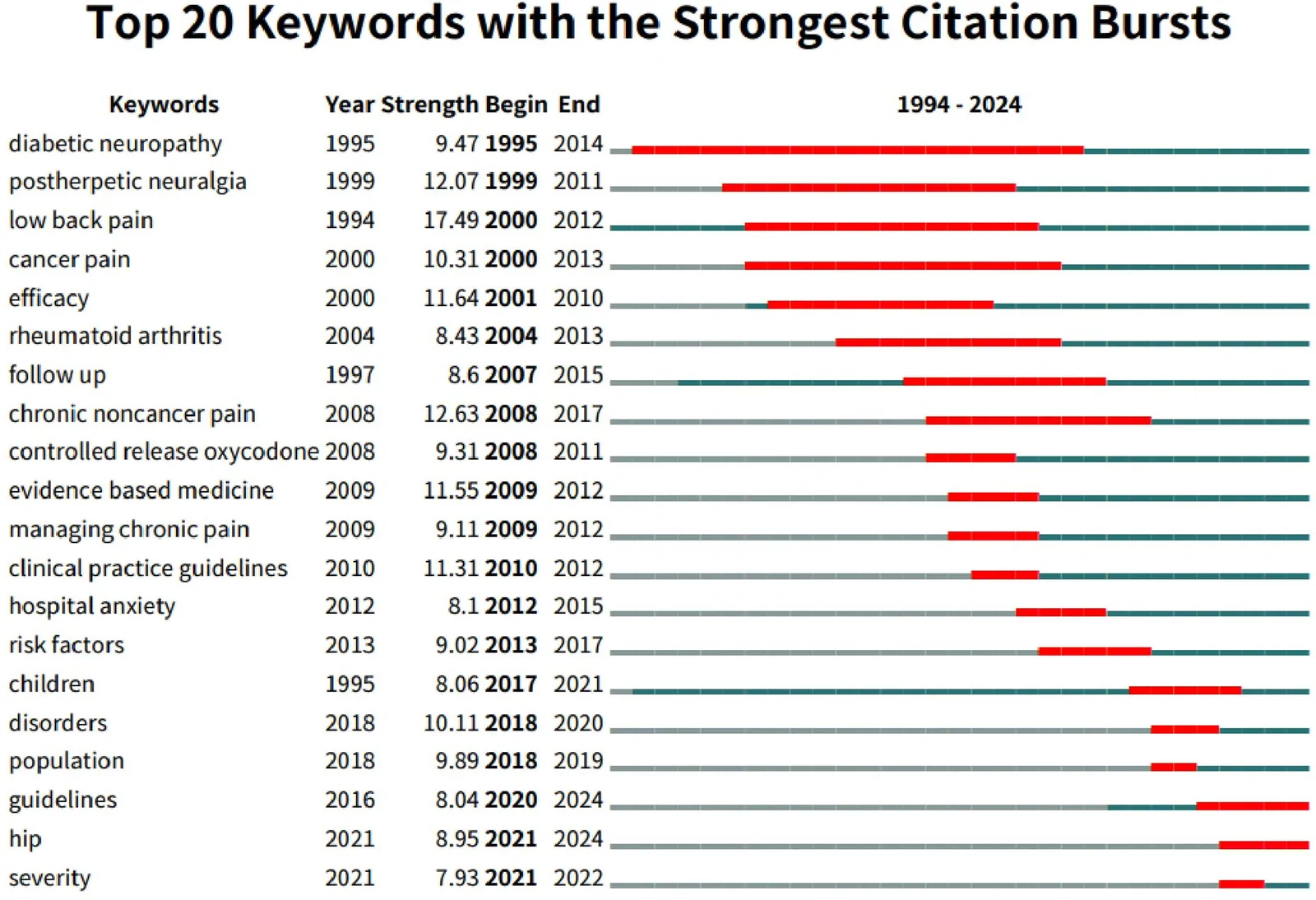
Top 20 keywords with the strongest citation bursts.

Furthermore, we employed keyword burst detection to identify research hotspots rapidly. The top 20 strongest keyword bursts from 1994 to 2024 are illustrated in Figure 8. The keyword “low back pain” exhibited the highest citation burst strength at 17.49. The longest sustained burst among these keywords was for “diabetic neuropathy,” lasting 19 years from 1994. Notably, “low back pain” has persisted since 1994 and continues to gain attention through 2024, with significant outbreaks from 2017 to 2021. Since 2017, several keywords, including “children,” “disorders,” “population,” “guidelines,” “hip,” and “severity,” have shown significant citation bursts, indicating a sustained focus on these topics in RCTs concerning chronic pain therapy research. Keywords such as “guidelines” and “hip” are expected to continue experiencing bursts until 2024, demonstrating ongoing interest in these areas.

## 4. Discussion

This bibliometric analysis provides a comprehensive overview of publication trends, research hotspots, and the evolving landscape of RCTs on chronic pain therapy from 1989 to 2024. The study highlights a significant increase in RCT publications during this timeframe, particularly noting a marked escalation since the late 1990s, culminating in a peak in 2022. This trend signifies a sustained research interest in chronic pain therapy and underscores the critical role of RCTs in the development of evidence-based therapeutic strategies.

### 4.1. General information

Our analysis reveals a significant geographic imbalance in chronic pain RCTs, with a predominant number of studies conducted in high-income countries, particularly the United States, the United Kingdom, and Canada. This concentration is primarily driven by disparities in research funding, regulatory efficiency, and trial infrastructure, rather than by disease burden.^[12]^ LMICs, despite having considerable unmet needs in pain management, contribute minimally to the trial landscape due to resource limitations and frequent early study discontinuations.^[13]^ This imbalance restricts global evidence equity and undermines the applicability of trial results to diverse populations. Additionally, studies conducted in high-resource settings often exclude clinically relevant subgroups, such as patients with comorbid depression or complex pain conditions, further diminishing external validity.^[14]^ Without structural investments in research capacity in LMICs and more inclusive trial designs, global pain management guidelines will continue to rely on an incomplete and geographically biased evidence base.

In terms of academic journals, *PAIN* is the most influential outlet, having served as the official journal of the International Association for the Study of Pain (IASP) since its inception. Its editorial mission prioritizes innovative, interdisciplinary pain research, and bibliometric analyses confirm its consistent leadership in citation metrics and trend-setting scholarship.^[15,16]^ Similarly, *Pain Medicine* and *The Journal of Pain* have established their niches by focusing on clinically oriented and behavioral health perspectives, respectively, and often publish landmark RCTs and guidelines. Their visibility and leadership roles within pain societies enhance their appeal to top-tier researchers.^[17]^

On the authorial side, R. Andrew Moore’s prominence is supported not only by his prolific output and citation volume but also by his methodological contributions to pain measurement and systematic review methodology. His leadership in Cochrane Pain, Palliative, and Supportive Care reviews has standardized evidence synthesis for pharmacologic interventions, influencing global interpretations of analgesic efficacy.^[18]^ His frequent collaborations with Derry Sheena and Manchikanti Laxmaiah further solidify a research cluster that is both quantitatively productive and methodologically influential.

The most frequently cited publications in this field include: the article titled “*Clinical Importance of Changes in Chronic Pain Intensity Measured on an 11-Point Numerical Pain Rating Scale*,” published in *PAIN* (IF = 5.9) in 2001, which has received 4038 citations.^[19]^ Farrar et al introduced an effective method for assessing pain intensity, emphasizing that even minor changes in patient-reported outcomes are significant. This work has transformed clinical practices in pain assessment. The second most cited article, “*Persistent Postsurgical Pain: Risk Factors and Prevention*,” published in the *Lancet* (IF = 98.4) in 2006, has garnered 2649 citations.^[20]^ This article highlighted persistent postsurgical pain, identifying risk factors and prevention strategies that have shaped both research and perioperative care. The third most cited publication is the “CDC Guideline for Prescribing Opioids for Chronic Pain – United States, 2016,” published in *JAMA* (IF = 63.1) in 2016, which has received 2042 citations.^[21]^ In this study, the authors issued the CDC guideline for opioid prescribing, marking a pivotal moment in pain management policy and clinical recommendations.

### 4.2. Research trends and hotspots

The keyword analysis indicates noteworthy shifts in research focus over the years. Among the examined topics, “low back pain” exhibited the highest total link strength. This aligns with global epidemiological data, which identifies low back pain as the leading cause of disability-adjusted life years worldwide.^[22]^ Its sustained research attention arises from both its population-level burden and its clinical complexity, which encompasses musculoskeletal, psychosocial, and neurological dimensions. Additionally, the COVID-19 pandemic has exacerbated chronic low back pain due to decreased physical activity, delayed access to care, and heightened psychological stress.^[23]^ These contextual factors have spurred an increase in biopsychosocial RCTs targeting low back pain with interventions such as CBT, mindfulness-based stress reduction, and interdisciplinary rehabilitation models.

### 4.3. Cluster analysis

The red cluster, centered around pain conditions such as “low back pain,” “cancer pain,” and “postoperative pain,” represents the biomedical core of the field. Research in this area traditionally focuses on pharmacological interventions, comparing analgesics, examining procedural efficacy, and preventing perioperative pain. This focus arises from the burden and complexity of these pain types – cancer pain, for instance, involves dynamic pathophysiology that necessitates rapid escalation strategies, while postoperative pain can lead to chronic pain if not managed appropriately from the outset. However, RCTs in this cluster struggle with standardizing endpoints across different pain etiologies, addressing ethical concerns (e.g., placebo use in severe pain), and accounting for heterogeneity in analgesic responses.^[24]^ Moving forward, the emphasis is shifting towards biotype-specific designs, such as differentiating between inflammatory and neuropathic subphenotypes, and integrating imaging or biomarker endpoints.

The green cluster focuses on psychological interventions, including “cognitive-behavioral therapy,” “mindfulness,” and “acceptance.” This reflects a biopsychosocial approach in pain medicine, recognizing cognitive appraisal, emotional regulation, and behavioral adaptation as not merely modifiers but also drivers of pain trajectories. The rise of acceptance and commitment therapy and mindfulness-based cognitive therapy in RCTs parallels evidence indicating that centralized pain often does not respond well to medications alone. However, trials in this cluster face challenges in maintaining treatment fidelity, ensuring therapist consistency, and adapting to cross-cultural or remote contexts. Engagement variability, particularly in low-literacy or high-stigma populations, remains a barrier to scalability. Emerging innovations such as digital CBT, AI-supported adherence feedback, and culturally tailored protocols offer potential solutions.^[25]^

The blue cluster emphasizes measurement and evaluation, capturing terms such as “risk,” “scales,” and “quality of life.” This domain reflects the field’s epistemic foundation – the tools used to validate interventions, stratify patients, and report outcomes. It emphasizes the ongoing push for core outcome sets in pain trials, which aim to standardize measurements across studies, thereby facilitating evidence synthesis and guideline formulation.^[26]^ Despite advancements, heterogeneity persists in pain intensity scales, measurement timing, and the inclusion of patient-reported outcomes. The future may involve dynamic assessment models that utilize ecological momentary assessment, wearable data, and adaptive algorithms to capture pain as a fluctuating, contextual experience rather than a static score.

The yellow cluster highlights alternative and complementary therapies, such as “acupuncture” and “osteoarthritis,” representing efforts to broaden therapeutic options beyond medications, often driven by patient demand and safety concerns. However, RCTs in this area encounter significant methodological challenges, including difficulties with blinding, high placebo responses, and the need for standardized protocols across diverse cultures and modalities.

Lastly, the purple cluster, though smaller, underscores long-term care and rehabilitation strategies through keywords such as “persistent pain” and “rehabilitation therapy.” For elderly patients or those with multiple comorbidities, managing persistent pain typically requires multidisciplinary, non-curative, and goal-oriented approaches. A best practice review indicates that geriatric populations particularly benefit from integrating pharmacotherapy with physical therapy, cognitive behavioral strategies, and vocational rehabilitation.^[27]^ Moving forward, incorporating long-term outcome measures – such as rehabilitation and care indicators – into chronic pain RCTs will be essential for ensuring clinical relevance. A paradigm shift is underway in pain management, recognizing pain not as a series of acute episodes but as a chronic condition necessitating long-term management.

### 4.4. Temporal evolution

From 2014 to 2016, keywords such as “double blind,” “placebo,” and “clinical trial” dominated, highlighting a focus on internal validity, rigor, and pharmacologic control conditions. During this period, studies often emphasized symptom reduction as the primary outcome, reflecting the prevalence of traditional RCT methodologies. However, rising placebo response rates began to challenge the effectiveness of these conventional structures – especially in neuropathic pain trials, where the placebo effect accounted for up to 75% of observed responses in certain groups.^[28,29]^ This trend not only threatened drug development but also prompted a reevaluation of RCT design priorities.

From 2016 to 2018, a conceptual shift became evident, with keywords such as “therapy,” “depression,” and “meta-analysis” indicating that the field began to address the complexity of chronic pain as a biopsychosocial phenomenon. This shift was supported by accumulating evidence that psychological distress, including depression and anxiety, is both a cause and a consequence of persistent pain. Meta-analyses during this period reinforced the significance of multidisciplinary and nonpharmacological strategies, with CBT emerging as a foundational component of RCTs.^[30]^

From 2018 onward, the introduction of keywords such as “acceptance,” “commitment therapy,” and “cognitive-behavioral therapy” reflects a deeper engagement with patient-centered paradigms. These therapies not only saw increased testing but also underwent greater scrutiny regarding their mechanisms and contexts of efficacy. For instance, single-case studies and idiographic methodologies were introduced to address treatment response heterogeneity and the limitations of group-level RCT inferences.^[30]^ The shift toward idiographic and ecologically valid methods, such as daily self-monitoring and digital CBT, underscores a growing alignment with personalized medicine principles.

### 4.5. Burst analysis

Burst analysis further highlights the evolving research focus over time. The sustained emphasis on keywords such as “guidelines” and “hip” throughout 2024 indicates a broader trend toward establishing evidence-based protocols in chronic pain management. The continued focus on “guidelines” reflects a persistent concern about the gap between evidence generation and real-world practices. Despite numerous RCTs evaluating pharmacologic and nonpharmacologic treatments for chronic pain, their findings often fail to translate into consistent, accessible, and equitable care. Recent studies reveal that guideline-concordant pain management remains underutilized, particularly in primary care and low-resource settings.^[31]^ Additionally, outdated or narrow guidelines may not adequately accommodate patient diversity or comorbid conditions.^[32]^ Therefore, future RCTs must increasingly be designed with guideline translation in mind, incorporating outcome frameworks, addressing implementation barriers, and considering economic modeling.

The keyword “hip” signifies a growing focus on site-specific and population-targeted pain conditions, particularly hip osteoarthritis and related degenerative disorders. As populations age and life expectancy increases, hip-related pain has become a major contributor to disability and healthcare costs. Recent RCTs examining physical therapy, telerehabilitation, and integrative models for hip pain demonstrate both efficacy and feasibility, especially in underserved or rural areas.^[33]^ However, challenges remain; hip pain patients often present with complex multimorbidity, necessitating individualized care plans while facing limited access to multidisciplinary services.

Together, these 2 keywords reflect the field’s shift towards a standardized yet stratified approach – adapting evidence-based treatment protocols and management guidelines to specific patient populations, clinical contexts, and cultural settings. Looking ahead, the field of chronic pain and RCTs is expected to evolve across multiple dimensions: hybrid trial designs that integrate efficacy and implementation research; personalized protocols tailored to specific conditions (particularly age-related joint disorders); and the establishment of industry-standard guidelines that employ equity-focused frameworks to address disparities in treatment access and therapeutic response.

### 4.6. Strengths and limitations

Our study presents several significant findings. First, it constitutes the first bibliometric analysis to evaluate 35 years of research on chronic pain therapy globally. While prior bibliometric studies in related fields have mapped research outputs and trends, our analysis distinctly concentrates on the landscape of RCTs in chronic pain therapy. Unlike earlier reviews that prioritized clinical outcomes or systematic reviews, this study provides a detailed visualization of publication patterns, elucidating gaps in geographic distribution and identifying emerging research priorities. Second, we offer comprehensive information regarding the countries, authors, institutions, and journals with the highest publication outputs. However, it is important to acknowledge potential biases, such as our reliance on citation counts, which may not fully capture an article’s clinical impact. Furthermore, the exclusion of non-English publications may constrain the scope of our analysis.

## 5. Conclusion

This study conducts a bibliometric analysis of literature related to RCTs on chronic pain therapy from 1989 to 2024. It evaluates global research trends, identifies key contributors, and examines the primary factors influencing this field. The analysis highlights research hotspots linked to keywords such as “low back pain,” “management,” and “quality of life,” while emerging areas of interest include “guidelines” and “hip.” Future research is expected to focus on developing standardized guidelines and addressing specific needs within diverse patient populations. Additionally, the implementation of large-scale, multi-center RCTs is anticipated to improve the quality of future studies. This research provides a comprehensive overview of global trends and hotspots in the field, offering valuable insights for researchers exploring potential future directions.

## Author contributions

**Data curation:** Meng Yang, Liuxue Lu, Yingge Tong, Qingye Liang, Qiuhuan Huang, Yuhai Yang, Hanxiang Li.

**Formal analysis:** Meng Yang, Liuxue Lu, Qingye Liang, Qiuhuan Huang, Yuhai Yang, Hanxiang Li.
